# Commentary: Immunogenicity of dupilumab in adult and pediatric patients with atopic dermatitis

**DOI:** 10.3389/fimmu.2026.1725709

**Published:** 2026-01-20

**Authors:** Miao-Chun Tsai, Hao-Yun Chen, Jhe-Cyun Liao, Yan-Yu Liu, Xiao-Ling Liu, Su-Boon Yong, Chia-Jung Li

**Affiliations:** 1 Department of Traditional Chinese Medicine, China Medical University Hospital, Taichung, Taiwan; 2 School of Medicine, China Medical University, Taichung, Taiwan; 3 School of Chinese Medicine, China Medical University, Taichung, Taiwan; 4 Department of Allergy and Immunology, China Medical University Children’s Hospital, Taichung, Taiwan; 5 Research Center of Allergy, Immunology and Microbiome (A.I.M.), China Medical University Hospital, China Medical University, Taichung, Taiwan; 6 Department of Medicine, College of Medicine, China Medical University, Taichung, Taiwan; 7 Department of Obstetrics and Gynecology, Kaohsiung Veterans General Hospital, Kaohsiung, Taiwan; 8 Institute of Biopharmaceutical Sciences, National Sun Yat-sen University, Kaohsiung, Taiwan

**Keywords:** anti-drug antibodies (ADA), atopic dermatitis (AD), dupilumab, immunogenicity, pediatric admission, pharmacokinetics

We read with great interest the report by Kamal et al. on the immunogenicity of dupilumab in patients with atopic dermatitis (AD) across age groups (1). Their pooled analysis of nine randomized controlled trials and extension studies provides important insight into anti-drug antibody (ADA) and neutralizing antibody (nAb) development in infants, children, adolescents, and adults. The authors conclude that immunogenicity exerts minimal impact on drug concentrations, efficacy, and safety, with clinically meaningful effects limited to a small subset of patients with high-titer ADAs. While this work is a valuable contribution, several methodological and interpretive issues warrant closer consideration.

## Pediatric confounders not fully addressed

As pediatric clinicians, based on real-world experience, we note that this analysis may benefit from more thorough consideration of key pediatric factors known to influence immunogenicity, such as weight-based dosing and concurrent use of topical immunomodulators. Pediatric pharmacology studies suggest that body weight–adjusted dosing and developmental immune differences may influence drug exposure and immune responses; however, whether these factors translate into clinically meaningful differences in immunogenicity remains to be confirmed in systematic investigations (2). In the Kamal dataset, treatment-emergent ADA rates ranged from 16.0% in adolescents to 2.0% in infants, but no stratified or multivariable modeling was performed for dose per kilogram, pubertal immune maturation, or topical exposure. Without such adjustments, the safety profile in children may be less precisely characterized, particularly for small subgroups with rare or delayed ADA responses. It would be useful for clinicians and patients if future studies could apply propensity score matching, mixed-effects modeling, and interaction analyses, and extend follow-up to detect delayed ADA emergence observed with other monoclonal antibodies (3, 4). In practice, such models would likely be feasible only in adult and adolescent cohorts, focusing on repeated measures of ADA titers (predominantly low-titer) as time-varying covariates, with random effects to account for inter-individual variability in exposure and disease activity. We acknowledge that, given the low incidence and low titers of ADA, these approaches may refine interpretation rather than materially alter overall conclusions.

## Reliance on descriptive statistics

The investigators relied primarily on descriptive summaries, which cannot disentangle relationships among ADA formation, pharmacokinetics, and clinical outcomes. Inferential approaches—such as multivariable mixed-effects models or Cox regression—would allow adjustment for disease severity, drug exposure, and concomitant therapies, thereby quantifying the independent impact of ADA titers. Prior biologic studies have shown that such modeling can reveal clinically significant ADA–efficacy associations that descriptive analyses overlook (5, 6). Consistent with prior reports in inflammatory skin disease and other immune-mediated conditions, anti-drug antibody formation has generally been associated with limited or no clinically meaningful impact on treatment efficacy when antibody responses are low-titer and transient. Incorporating time-to-event analyses and exposure–response frameworks, rather than formal dose–response modeling, may help clarify whether ADA emergence precedes changes in pharmacokinetics or efficacy.

## Incomplete interpretation of adolescent ADA rates

The suggestion that higher ADA incidence in adolescents reflects predominantly transient IgM responses provides a plausible explanation, but may oversimplify the underlying biology. Adolescence is characterized by immune remodeling, pharmacokinetic variability, and hormonal and microbiome-associated changes that have been hypothesized to influence immunogenicity (7–9). At present, however, evidence linking these factors directly to clinically meaningful alterations in dupilumab pharmacokinetics or efficacy remains limited.

Mechanistic studies incorporating age-stratified pharmacokinetic analyses and immunophenotyping may help clarify the biological basis of transient ADA responses in adolescents, while acknowledging that available clinical data to date consistently indicate minimal impact on treatment efficacy.

## Conclusion

In summary, the available evidence supports the conclusion that dupilumab immunogenicity has minimal clinical impact across age groups, consistent with the findings reported by Kamal et al. At the same time, the reliance on descriptive analyses reflects limitations in data availability rather than analytical oversight. Longer-term follow-up, transparent access to de-identified patient-level data, and selected mechanistic studies may help refine understanding of rare or delayed immunogenicity signals, particularly in pediatric populations with prolonged exposure. Such efforts are likely to enhance precision and durability of treatment without fundamentally altering the established safety and efficacy profile of dupilumab.
